# A Process Account of Dehumanization: Extending the Framework with a Developmental Research Agenda

**DOI:** 10.3390/bs16060867

**Published:** 2026-05-29

**Authors:** Jeroen Vaes, Cecilia Dapor, Federica Meconi, Ermanno Quadrelli, Elisa Roberti, Daniela Ruzzante, Alessia Testa

**Affiliations:** 1 Department of Psychology and Cognitive Science, University of Trento, 38068 Rovereto, Italy; 2 Department of Psychology, University of Milano-Bicocca, 20126 Milan, Italy; 3 NeuroMI, Milan Center for Neuroscience, 20126 Milan, Italy

**Keywords:** dehumanization, mentalizing, intergroup relations, developmental processes, neuroscience

## Abstract

In the last 25 years, research on dehumanization—the tendency to perceive others as less than fully human—has spiked and evolved in many ways. In the current review, we will provide an overview of the various methodological and conceptual trends in this research area and introduce a new way of conceptualizing dehumanizing perceptions. Focusing on developments in neuroscience that have shown how human and object stimuli are typically processed in different ways using specific brain areas, dehumanization can be understood as the fading of this human–object divide. We will demonstrate what this process account of dehumanization implies for the understanding of the concept, how it can respond to some of the recent controversies and critiques, and how a research agenda integrating the study of developmental mechanisms can bolster our understanding of dehumanization processes.

## 1. Introduction

Arguably, dehumanization—the tendency to see others as less than fully human—has always existed. From when the first relics representing the other were found, instances of dehumanization have been reported. For example, in royal inscriptions in which defeated groups were portrayed as uncivilized (see for example the common interpretation of the victory stele of Naram-Sin 2250 BC, Victory Stele of Naram-Sin, 2025). More widely documented is the barbarian concept by which non-Greeks “Barbaroi” were portrayed as irrational and uncivilized in ancient Greece. The Aristotelian concept of “natural slaves” that he coined around the 4th century BC, described foreigners who lack full rational capacity and therefore the ability to rule themselves, allowing them to be enslaved “for their own good” (see Stuurman, 2021, for a recent discussion).

Even though the term “dehumanization” only appeared in the English dictionary around the turn of the 19th century, the phenomenon has a long history and contemporary examples abound. Given its prevalence, the current review aims to give an overview of the concept and the way it has been studied in social psychology. In doing so, it will focus on the neural and cognitive processes that are involved in dehumanizing perceptions and how the process of dehumanization can be further understood through a research agenda that integrates the study of developmental mechanisms. This process account of dehumanization will further clarify the concept and allow us to respond to a set of recent criticisms and controversies.

## 2. What Is Dehumanization?

Research and theorizing on dehumanization in social psychology has gained momentum at the beginning of this century. Infrahumanization theory (J.-P. Leyens et al., 2000) and Haslam’s dual model of dehumanization (Haslam, 2006) both contributed in framing dehumanization as a general phenomenon of social judgment that not only occurred in contexts of extreme violence and intractable conflict. They defined dehumanization as the tendency to see others as lesser human beings, or as less than fully human. This work gave way to a research program that showed the relevance of this concept across intergroup (Vaes et al., 2012) and interpersonal relations (Karantzas et al., 2023), the sexual objectification of women (Pecini et al., 2023; Vaes et al., 2014), doctor–patient (Haque & Waytz, 2012; Vaes & Muratore, 2013), and human–AI interactions (Dang & Liu, 2025) to name but a few.

This new perspective on dehumanization has characterized the concept according to some key features (P. G. Bain et al., 2013; Haslam & Loughnan, 2014; N. S. Kteily & Landry, 2022; Vaes et al., 2021). Firstly, while other accounts defined dehumanization as a categorical phenomenon in which a human essence is either given or denied (Smith, 2011), the new look on dehumanization has acknowledged it to be a graded or incremental process. As a result, not all human abilities or capacities need to be denied to another person to be dehumanized. It is perfectly possible that a dehumanized target still possesses some human characteristics, but maybe less so than others or compared to the same person in a different context. Therefore, dehumanization is often better understood in a relative way (Vaes et al., 2021). Furthermore, dehumanization does not need to be literal, to be real. Other phenomena of social perception (e.g., stereotypes) exert their influence regardless of whether they are true or not (Dixon, 2017; Schneider, 2005). To dehumanize or feel dehumanized a person does not have to be literally perceived as an animal or an object. Finally, even though dehumanization often occurs towards strongly disliked targets, it can be conceptualized and measured independently from mere dislike. Indeed, dehumanization has been reported towards liked targets (e.g., sexualized women, Vaes et al., 2011) and explains unique variance over and above evaluative judgments in helping behavior (Andrighetto et al., 2014) and violence (Landry et al., 2026) among other behaviors.

## 3. How Do We Dehumanize?

Dehumanization can take many forms. Previous reviews on the concept (Haslam & Loughnan, 2014; N. S. Kteily & Landry, 2022) have taught us that dehumanization can be subtle or blatant, implicit or explicit and occurs on different dimensions of humanness. The latter distinction is central in Haslam’s dual model of dehumanization (Haslam, 2006). In this model, animalistic dehumanization is defined by the contrast between humans and animals and occurs whenever people are directly associated with animals or denied uniquely human attributes, such as civility, refinement, moral sensibility, rationality, and maturity. Mechanistic dehumanization, instead, contrasts humans with inanimate objects or machines and follows when people are directly associated with objects or denied human nature attributes such as emotionality, warmth, cognitive openness, agency, and depth.

These various expressions of dehumanization have been assessed with a variety of measures. Arguably, the most common measures of dehumanization can be defined as characteristic-based. These measures use some attribute or characteristic that expresses a central element of what it means to be human, e.g., secondary emotions (J. Leyens et al., 2001), human nature or uniquely human characteristics (Haslam, 2006), and agency or experience traits (H. M. Gray et al., 2007) and gauge the extent to which these characteristics are denied or granted to some social targets. In a similar vein, dehumanization measures have used machine-like (Loughnan & Haslam, 2007) or animal (Goff et al., 2008) imagery and assessed the extent to which these images are associated to social targets. The use of metaphorical images has led to the development of a popular measure that uses the historic image of human evolution that depicts an ape walking on four hands on the left, all the way to the homo sapiens, who walks erect on the right side of the image. The Ascent of Man scale (N. Kteily et al., 2015) then requires participants to locate social groups on this image to indicate how human or evolved they think the targets are. Although they use a different response format, metaphor- and characteristic-based measures of dehumanization are often highly correlated (Loughnan et al., 2009), suggesting that they tap into similar concepts. In the case of the Ascent of Man scale, the task is reminiscent of attributing—or denying—to others a degree of highly evolved cognitive or mental capacities.

These more explicit, and often blatant measures of dehumanization contrast with more subtle forms that link dehumanization to basic perceptual mechanisms. The most straightforward version of these measures presents an animal or doll-like face that gradually morphs into a fully human face and asks participants to press a key when the stimulus appears human to them. The assumption underlying these paradigms is that participants are not only recognizing a physically human face, but also detect the emergence of life or a mind. This assumption has been confirmed, showing that the point of recognition can be shifted, leading to perceptions of greater humanity when facial features that typically convey the presence of a mind, such as a person’s eyes, are emphasized (Looser & Wheatley, 2010). Adaptations of this paradigm further show that the tipping point at which humanness is perceived can be influenced by higher-order variables, such as group membership. Specifically, ingroup faces tend to require a more fully human appearance before they are perceived as such, compared to outgroup faces (Boccato et al., 2007; Goff et al., 2008; Krumhuber et al., 2015).

Similar work examined whether observers engage in configural face processing when confronted with dehumanized targets (Fincher et al., 2024; Hugenberg et al., 2016). Configural face processing is known to be crucial for the correct recognition of human faces and bodies, given that any manipulation, such as inverting a face or body, that disrupts this process reliably impairs the identification of these stimuli while leaving the recognition of objects intact. Markedly, dehumanized targets are not affected by these manipulations. Inverting depictions of sexually objectified women (Bernard et al., 2012) or the faces of alleged criminals (Fincher & Tetlock, 2016) did not affect their recognition, as if they were objects without the use of configural processing. In a similar vein, inverted human faces are attributed fewer human traits, further indicating that the interruption of configural processing is linked to dehumanized perceptions (Hugenberg et al., 2016).

Extending these findings, work in neuroscience has suggested that brain networks central to social cognition are often not activated when perceiving or interacting with dehumanized groups (see Harris & Delgado, 2025, for a review). The medial prefrontal cortex (mPFC), typically implicated in elaborating social targets, shows reduced activation toward groups that are judged low in both warmth and competence, such as drug addicts or homeless individuals (Harris & Fiske, 2006). Similarly, hostile sexist men show diminished mPFC responses when viewing sexually objectified women (Cikara et al., 2011). Other research directly compared the way human and perceptually similar mindless objects were elaborated interpreting the lack of distinction or partial overlap of the way these stimuli would be processed as an indication of dehumanization. Vaes et al. (2019) measured participants’ neural reactions in the well-known odd-ball paradigm (Squires et al., 1975). Within this paradigm, the infrequent stimulus (i.e., the oddball) is known to trigger a positive ERP that peaks between 250 and 500 ms, the P300, and its amplitude increases the more the oddball is seen as deviant (see Ghani et al., 2020, for a review). In a first study, the frequently presented targets were objectified (i.e., scarcely dressed) male and female targets that were infrequently interrupted with gender-matched, perceptually similar doll-like avatars that were construed and based on the frequent human stimuli. As such, decrements in the P300 amplitude of the oddball can be interpreted as an increase in the overlap between the way mindless, doll-like and mindful human stimuli are elaborated. As expected, relative to the frequent pictures, all infrequent stimuli activated an increase in the P300 amplitude, but less so for the objectified women compared to their male counterparts. This result was confined to objectified depictions of women, as non-objectified female and male human targets were equally and clearly differentiated from the doll-like objects (Vaes et al., 2019). Using a similar paradigm, Ruzzante and Vaes (2023) extended these findings to other social targets that are potentially dehumanized such as outgroup members (Ruzzante & Vaes, 2021) and inverted faces (Ruzzante & Vaes, 2024a).

Taken together, these various methodologies have documented processes of dehumanization towards ethnic and national groups (P. Bain et al., 2009; Vaes & Paladino, 2010), immigrant and refugee groups (Esses et al., 2008; Hodson & Costello, 2007), occupational groups (Iatridis, 2013; Loughnan & Haslam, 2007), people from lower social class (Loughnan et al., 2014), people with physical (Vaes & Muratore, 2013) or mental health issues (Martinez et al., 2011), women who are sexualized (Vaes et al., 2011), people who have committed violent crimes (Bastian et al., 2013), and people who identify as asexual (MacInnis & Hodson, 2012), to name but a few. The fact that the same targets or the same conditions created comparable levels of dehumanization across a variety of measures has yielded convergent validity for this line of research (Campbell & Fiske, 1959). At the same time, research has demonstrated that dehumanization biases cannot be reduced to simple dislike or intergroup prejudice, thereby establishing divergent validity as well (Bruneau et al., 2018; Vaes, 2023).

## 4. Why Do We Dehumanize?

The variety and prevalence with which dehumanization can be observed beg the question of why people dehumanize fellow human beings. Rather than explaining the specific processes that drive dehumanization in specific contexts, several more general theories have been formulated that might explain why people engage in such, typically destructive, thinking. Without wanting to be exhaustive, we review three of these theories here: one that focusses on evolutionary mechanisms, a second that emphasizes cultural and existential drives, and a third one that centers on instances in which dehumanization can be functional.

The human self-domestication hypothesis states that modern humans were selected for prosociality (Hare, 2017; Hare & Woods, 2020). This selection process is apparent in our morphology and physiology that evolved over time. For example, humans reduced their brow ridge and increased their facial length, making them look more gentle (Cieri et al., 2014). In addition, the occurrence of white sclera increased the capacity of humans to use eye gaze as an important communicative instrument (Tomasello et al., 2007). These more gentle physiological characteristics evolved together with higher order social cognitive capacities related to mentalizing. Indeed, our ability to reason about the minds of others allows us to cooperate, imitate, deceive, and learn from one another (Tomasello, 2009). Similar physiological and morphological changes and the acquisition of comparable unique socio-cognitive skills can be found in animals that self-domesticated, such as dogs compared to wolves or bonobos compared to chimpanzees (see Hare, 2017, for an overview). These unique mentalizing skills have allowed humans to coordinate and cooperate within larger groups, making them stronger and more effective in fending off enemies or outgroups. Indeed, according to this perspective, the uniquely human mentalizing skills that facilitate cooperation among ingroup members allow us to dehumanize those we consider too dissimilar from us. Cooperation becomes costly when it is extended indefinitely because the likelihood of reciprocation decreases. This supports the idea that social cognition and mentalizing evolved specifically to support life within groups (Caporael, 1997). The strong link between our capacity to empathize and dehumanize has been demonstrated within adults by manipulating the neuropeptide oxytocin. Oxytocin is known to promote social bonding and empathy in humans, but also increases ingroup favoritism. Adults who were administered oxytocin humanized the ingroup more compared to the outgroup and saw them in a more positive light (De Dreu et al., 2011).

Cultural and existential drives have been forwarded to explain dehumanization as well. Terror management theory (TMT; Greenberg et al., 1997) states that the uniquely human combination of having a strong instinct for survival and a constant awareness of the inevitability of death gives rise to an existential terror. Humans manage this existential fear by creating and embracing cultural worldviews and by believing that they live up to the values of these cultural systems. Cultures provide a shared conception of reality that gives structure and meaning to its members. Such beliefs provide a subjective feeling of safety and death transcendence that have an anxiety-buffering function against the existential fears surrounding our vulnerability and mortality. Goldenberg et al. (2000) and Goldenberg (2005) extended this framework to the more physical and animalistic aspects of our existence. Our bodies get tired, sick, stink, excrete, directly reflect our basic impulses, and in doing so emphasize our animal nature and our vulnerability to death. An abundant amount of research has demonstrated that in order to minimize this existential threat, people not only deny their similarity to animals but also engage in strategies that allow them to emphasize the uniquely human aspects of the self and of the groups they belong to (Goldenberg et al., 2001, 2009). Vaes et al. (2010) tested this directly, demonstrating that when people were asked to think about their own death, they endowed their ingroup with more humanness compared to an equally aversive, but death-unrelated control condition.

Yet another framework reveals the potential functional role of dehumanization. Dividing groups in terms of humanity has shown to help us to organize the world and decide who can be given more or less moral regard. For centuries, philosophers, theologians and early natural scientists ranked living entities hierarchically in the great chain of being. Morally questionable entities such as demons, devils, and animals were typically found on the bottom, whereas humans, saints and gods were located in the moral top-half of the chain (Lovejoy, 1964). Even though modern science explicitly distanced itself from such rankings after the second world war, a similar framework has persisted in the way lay theories organize the moral universe. This has led Brandt and Reyna (2011) to introduce the social cognitive chain of being that states that ordinary people continue to represent morality on a vertical continuum that ranges from the most immoral and evil at the bottom to the most virtuous and good social targets at the top. Interestingly, perceiving others as less human or ascribing human qualities or mental states to nonhuman entities or gods allows them to move down or up the chain of being, respectively. As a result, the amount of humanness we perceive in others becomes an organizational principle that divides the world in those who deserve moral regard or possess moral agency and those who do not (see K. Gray & Wegner, 2009; N. S. Kteily & Landry, 2022, for a similar reasoning).

Apart from these more distal and fundamental drives, more specific motives have been unveiled in the contexts in which dehumanization occurs (see for example, dehumanization in the work place, Baldissarri et al., 2022; medical dehumanization, Haque & Waytz, 2012; sexual objectification, Pecini et al., 2023; intergroup dehumanization, Vaes et al., 2012). Taken together, these three perspectives clearly suggest how evolutionary, cultural and functional dynamics underlie processes of dehumanization highlighting the centrality of this process in our daily lives (Harris & Delgado, 2025).

## 5. A Process Account of Dehumanization

From the literature we have reviewed so far, one might get the impression that dehumanization implies forming an attitude in human terms towards others. This attitude can be explicit or implicit, blatant or subtle, animalistic or mechanistic, and relatively less human compared to other targets or dehumanizing in absolute terms. This characterization of dehumanization has been central in previous reviews (Haslam, 2013, 2025; N. S. Kteily & Landry, 2022) and tends to overshadow the fact that (de)humanization is a central process in social perception that is rooted in our capacity to reason about other people’s minds. Arguably, mentalizing—inferring or attributing mental states to others—is essential for perceiving others as human. Let us therefore unpack the process of mentalizing and how it relates to research on dehumanization.

The course and outcome of our interactions among humans depends on our capacity and willingness to infer their thoughts, intentions, desires, and attitudes. In other words, our capacity and willingness to reflect and reason about their mind. Such mentalizing processes are completely absent when interacting with unambiguous objects that are not anthropomorphized in any way. The uniqueness of human-specific processing has been confirmed in neuroscientific research that has demonstrated how certain brain areas and networks are uniquely engaged in our interactions with human, but not object stimuli (Mitchell et al., 2002). This specialization for human stimuli is not only perceptual, but involves neural networks that are involved in social cognition, empathy, and moral decision making (see Harris & Delgado, 2025, for a recent review).

Our capacity to reason about other people’s minds is the result of the interaction between bottom-up processes that only take the low-level, feature-based sensory input of the stimulus into consideration and top-down processes that stem from the perceiver’s past experiences and knowledge, their motivations and goals and the broader context (Meconi et al., 2021; Sessa et al., 2014). Therefore, mind denial can be the result of a lack of willingness or our incapacity to infer other people’s minds or the application of previously acquired knowledge that does not allow us to fully see the mind of others. In favor of this distinction, purely perceptual manipulations such as the facial-width-to-height-ratio (FWHR; Deska et al., 2018), the facial inversion effect (Fincher et al., 2024), or a specific emphasis on a person’s eyes (Looser & Wheatley, 2010) have shown to moderate the extent to which we ascribe or deny mental attributes to others. These manipulations only change low-level, sensory based stimulus information, without conveying meaning per se. On the contrary, a large number of contextual variables have shown to influence the extent to which we imbue others with a mind. Probably the most frequently studied contextual variable—in which a person’s previous knowledge, experience and motivation play a crucial role—is group membership. Ingroup members are typically seen as more human and mindful than outgroup members (P. Bain et al., 2009; Hackel et al., 2014; Krumhuber et al., 2015; J. Leyens et al., 2001).

**Figure 1:**
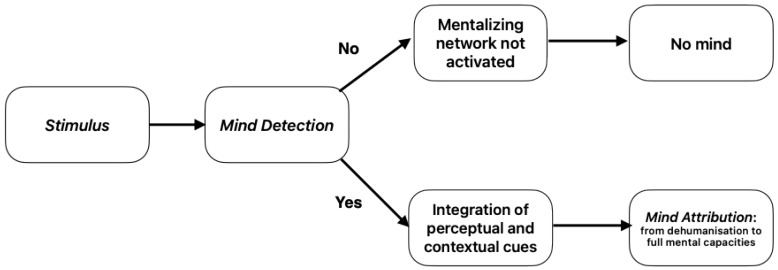
The two-phase model of mentalizing.

Furthermore, mentalizing can be divided into two temporarily distinct phases. Just as one cannot suppress an unidentified thought (Wegner, 1994), one cannot infer a mind that has not first been detected (Zaki, 2014). Before attributing mental capacities to others, people must determine whether a mind is present at all. Applied to mentalizing, this suggests an initial on–off mechanism that activates or deactivates the mentalizing network and the associated higher-order cognitive processes. If no mind is detected—as with unambiguous and inanimate objects such as floor tiles or a coffee cup—mentalizing does not occur. Instead, when the stimulus contains minimal human or animacy cues, a mind might be detected. However, this does not guarantee the attribution of full mental capacities. When a mind is detected, mentalizing is engaged and mental capacities are attributed gradually. This occurs in the subsequent mind attribution phase where perceptual and contextual information are integrated to shape judgments of others’ mental capacities (see Figure 1 for a schematic overview). Thus, like dehumanization, mind denial requires prior mind detection, followed by the downregulation of attributed capacities. This process mirrors the paradox of dehumanization (Phillips, 2022; Smith, 2016): when we dehumanize others we regard them as human and subhuman at the same time, since denying humanity presupposes its initial recognition. In a similar vein, anthropomorphism requires an initial recognition that a non-human entity might have a mind before a mind can be attributed to a certain degree.

This two-phase model was tested by adapting the experimental procedure of Vaes et al. (2019) into the (de)-mentalization oddball paradigm (D-MOP, Ruzzante & Vaes, 2023). This time, the infrequent, oddball stimuli were doll-like faces that were created by morphing the frequently presented human faces (30%) with a doll-face (70%). In a total of four EEG studies, participants were asked to categorize these stimuli as humans or avatars while both perceptual (i.e., FWHR; Ruzzante & Vaes, 2021; the inversion effect; Ruzzante & Vaes, 2024a) and contextual variables (i.e., comparing neural reactions toward ingroup and outgroup targets) were manipulated. The two phases of mentalizing were expected to correspond with two specific event-related potentials (ERPs). The first, the N170, is an early negative wave that peaks around 170 ms after stimulus onset. This ERP was thought to overlap with the mind detection phase, given that the N170 is especially triggered when viewing human faces compared to objects, animal faces or body parts (Bentin et al., 1996). In a similar vein, results of all studies confirmed that the N170 was the first moment in which mindful, human and mindless, doll-like avatars were differentiated for the first time. This effect occurred regardless of any of the perceptual or contextual manipulations of the facial stimuli.

The mind attribution phase, instead, was expected to overlap with the P300. It was expected that mind denial would correspond with a less pronounced oddball effect suggesting that the mindless doll-like targets were elaborated similarly to the mindful, human targets. In almost all studies, the outgroup faces elicited a smaller oddball effect than those of the ingroup. This effect correlated with an implicit, behavioral measure of mind attribution, reinforcing the interpretation of these results in terms of mentalizing. In the only instance in which group membership did not moderate the P300, the inverted faces that are known to trigger dehumanized perceptions (Hugenberg et al., 2016) showed a smaller oddball effect compared to upright faces (Ruzzante & Vaes, 2024a). Likely, presenting the faces upright or inverted was more salient to participants than an ingroup–outgroup label that was only presented at the beginning of each experimental block. In two of the four studies, the perceptual variable moderated the group membership effect, suggesting that perceptual and contextual variables interact in the mind attribution phase to attribute a mind to the target.

Overall, these findings demonstrate the dynamic progression of mentalizing, distinguishing between a mind detection phase that discerns whether a mind can be detected and the mind attribution phase in which both perceptual and contextual variables are integrated to determine the extent to which the target has a mind.

## 6. Responses to Critiques and Challenges on Dehumanization

The process account introduced above embeds the research on dehumanization within our capacity to reason about other people’s minds. In doing so, it has no ambition to substitute other frameworks of dehumanization, but rather to start reasoning about the neural, cognitive and affective processes that underlie dehumanized perceptions. We believe that this process account of dehumanization has several advantages, among them its capacity to counter a series of challenges and critiques that have been directed at dehumanization research.

### 6.1. Dehumanization and the Broader Valence Dimension

First of all, this process account resolves some of the ambiguities that became apparent using characteristic-based measures of dehumanization. These measures typically identify a series of traits or characteristics that are pre-tested to represent what it means to be human and then attributed to one or more social targets. The less these traits are attributed to the target, the more the target is dehumanized. This type of measure becomes problematic when the context or the inherent characteristics of the target provide other reasons for attributing these traits rather than their humanness. For example, when comparing the attribution of a set of human traits between adults and children, the latter would be attributed less of these traits. However, given the fact that these measures often contain references to higher cognitive processes (e.g., being educated, rational, emotionally responsive) that one attains throughout the developmental process, one can doubt that the difference between adults and children actually indicates a dehumanized perception. A similar reasoning can be applied to social categories, such as people with intellectual impairments or mental health issues that might be affected by an able-centric bias in defining what it means to be human. A process account of dehumanization, instead, acknowledges the fact that we adapt our speech and words to convey certain ideas to children or individuals who might have a hard time understanding what we are saying as a sign of recognizing their full humanness.

In line with this first comment, several scholars have questioned the independence of dehumanization from a broader valence dimension (Lang, 2020; Over, 2021). Specifically, Over (2021) suggested that characteristic-based measures of dehumanization often omit clearly human, but antisocial attributes such as jealousy, dishonesty, and disloyalty. As a result, characteristic-based measures of dehumanization focus on prosocial traits and emotions that are more likely attributed to one’s ingroup, not because they are human, but because they are desirable to possess (Enock et al., 2021). Even though a lot of dehumanization research went to great lengths to demonstrate that their measures were not conflated with negative evaluations, Over (2021) exposes an important weakness of characteristic-based measures. Humanness is a broad and multifaceted concept that is difficult to capture in a small number of attributes. One can never fully exclude that additional human attributes, such as the ones Over mentions, would have tilted the outcome in the other direction. Therefore, it is important to pre-test the human attributes thoroughly in the specific context and towards the specific targets to ascertain that they can be used and interpreted appropriately. Alternatively, other work has not only measured the typicality of a larger set of traits but also the humanness of each characteristic and analyzed the correlation between both dimensions for each participant as an index of dehumanization (Vaes & Paladino, 2010). Directly replying to Enock et al. (2021), who did not find outgroup dehumanization when both pro- and antisocial traits were used, Vaes (2023) conceptually replicated a subset of their studies measuring both the typicality and the humanness of the same set of traits. The correlations between both dimensions clearly demonstrated the presence of outgroup dehumanization over and above intergroup prejudice. In addition, the methodologies that are discussed in the current manuscript, which use physiological parameters in paradigms that eliminate semantic labels and directly compare how mindful, human, and mindless object stimuli are processed when they represent members of different social categories, avoid this problem altogether.

### 6.2. The Paradox of Dehumanization

How can one reconcile the idea that dehumanization implies denying a person’s human status with the fact that dehumanization is a process that is only applied to people, not to animals or objects? This paradox has been mostly discussed for categorical formulations of dehumanization where a person completely loses one’s humanity and becomes the equivalent of an animal or object, and this contradiction clearly constitutes a problem (Smith, 2011). This paradox, however, does not pose a problem when dehumanization is defined as a graded process through which we perceive others as less than fully human, as we have proposed throughout this review. Such a framing recognizes that while multiple aspects of humanness may be denied to a target, it is possible that some other human characteristics remain intact. Moreover, the process account of dehumanization treats this paradox as a central part of the process. Given that one cannot infer a mind that has not been detected, mind denial will only occur when the potential for a mind has been identified. As such, a process account fully embraces the impossibility to dehumanize one who we have not seen as potentially human in the first place.

## 7. Research Agenda Testing Developmental Mechanisms in Dehumanization

The developmental agenda we propose is not intended as a stand-alone extension of the review, but as a direct extension of the process account outlined above. This account stresses the central role of social perception and mentalizing in dehumanization. Although these abilities have been widely studied from around 4- to 5- years of age onward, their early precursors can already be observed in infancy (Poulin-Dubois et al., 2023; Rakoczy, 2022). Therefore, the first years of life are crucial to understanding how brain network maturation and early experiences intertwine and lay the foundation of adult social cognition. From this perspective, the proposed developmental agenda builds on a broader literature on the early development of mind understanding, including work on theory of mind and its developmental foundations in infancy and early childhood (e.g., Rakoczy, 2022).

Yet, research on dehumanization across development is still scarce (McLoughlin & Over, 2018). Only a handful of studies has demonstrated that children as young as 6 years old reliably show a tendency to attribute more human traits (Costello & Hodson, 2014; Van Noorden et al., 2014) or uniquely human emotions (Chas et al., 2015; Martin et al., 2008) to their own group or friends compared to a range of outgroups (i.e., other race children, Costello & Hodson, 2014; non-friends, Van Noorden et al., 2014; minimal outgroups in competition, Zhou & Hare, 2022). A potential weakness of these studies is that they use the same human characteristics that were used in adult research, assuming that children have a similar understanding of humanness as adults (McLoughlin, 2023). To overcome this shortcoming, more recent research has adopted methods that do not rely on children’s comprehension of semantic stimuli. McLoughlin and Over (2017), for example, asked 5- and 6-year old children to rate the humanness of ambiguous human–doll faces, demonstrating that when these faces represented outgroup faces, they were judged as less human compared to those of ingroup members. In a similar vein, Zhou and Hare (2022) found that 5-to-12 year olds rated the outgroup as less human on a variety of pictorial measures, such as the Ascent of Man and the same face perception scale that McLoughlin and Over (2017) used.

Even though these studies provide converging evidence that dehumanization biases can be found in children, we believe that developmental research provides a unique opportunity to test some of the basic processes that underlie dehumanized perceptions. Specifically, the process account on dehumanization we illustrated above allows us to formulate a research agenda testing these developmental processes. On the one hand, it helps to delineate the emerging cognitive abilities and mechanisms that potentially drive dehumanized perceptions. On the other hand, it provides the methodologies that make it possible to directly compare the responses of children and adults.

One of the main assumptions of our process account is that dehumanization is rooted in our capacity to reason about other people’s minds. As a consequence, one can expect dehumanization to increase with children’s development of mentalizing abilities. This hypothesis was directly tested in two studies measuring the understanding of 3-to-6 year olds’ first- and second-order false beliefs and the dehumanization of others using pictorial measures in a minimal group paradigm (Zhou & Hare, 2026). Consistent with the idea that dehumanization is rooted in our capacity to reason about other people’s minds, only children that had some mentalizing skills rated the outgroup as less human than the ingroup. This effect was already observed in children who showed first-order false-belief understanding and became stronger among children who understood second-order beliefs. Interestingly, this pattern of results was unique for outgroup dehumanization and did not occur when measuring outgroup prejudice. Children consistently liked the outgroup less than the ingroup well before the emergence of belief attribution capacities. All these changes were observed after controlling for age and gender. These results strongly reinforce the idea that dehumanization and mentalizing skills are closely related, demonstrating that in order to be able to deny a mind, one needs to have the capacity to detect it in the first place. These findings also provide indirect support to our process model that states that dehumanization requires the detection of a mind (i.e., mind detection phase) before it can be denied (i.e., mind attribution phase).

More research, however, is warranted, especially studies on infants, to help establish the roots and mechanisms of the emerging cognitive abilities that underlie dehumanized perceptions. Even though full-blown mental state reasoning explicitly manifests around 5 years of age (Callaghan et al., 2005) and further develops throughout the life span (Yeung et al., 2024), infants are sensitive to many social cues. Their ability to differentiate animate from inanimate objects relies not only on biological properties and animacy, but also on psychological cues such as intentions and goal-directedness (Goldman & Poulin-Dubois, 2024). Among the earliest and most powerful of these cues is the eye gaze. For example, research has shown that 6- to 8-month-old infants’ attention is guided by the gaze of both humans and robots, but not of a non-humanoid object (Linnunsalo et al., 2024), suggesting that gaze may trigger socially oriented attention even in the absence of established conceptual knowledge about the agent, a humanoid robot programmed specifically to socially interact with people. Infants are also sensitive to biological and behavioral features that distinguish animate agents from inert objects and display early precursors of mentalizing capacities (Natale et al., 2014; Southgate & Vernetti, 2014). Indeed, infants are capable of human–object distinction (Legerstee et al., 2000), respond to people and inanimate objects differently, and attend to specific human-like features that serve to identify people, such as faces (Morton & Johnson, 1991; Turati & Quadrelli, 2017), and internal experiences (i.e., goals and desires, Brandone & Wellman, 2009). Furthermore, research suggests that ethnicity and intergroup biases emerge in infancy as well. One example is the well-known other-race effect, whereby both child and adult observers have greater difficulty telling apart faces from within other ethnicities compared to faces from within their own ethnicity. This effect takes root in infancy (Quinn et al., 2019; Sugden & Marquis, 2017). In addition, 9-month-old infants associate familiar own-ethnicity faces with positive emotional valence and unfamiliar other-ethnicity faces with negative emotional valence, thus suggesting a bias that may lead to later implicit ethnicity biases, favoring own- over other-ethnicity (Xiao et al., 2018). Therefore, research on 9- to-12-month-old infants could already help identify the necessary cognitive processes that underlie dehumanization.

Building on this developmental trajectory, we propose extending and adapting the D-MOP-paradigm using EEG combined with Fast Periodic Visual Stimulation (FPVS) to enable direct comparisons between infants and adults. FPVS is a sensitive, visual discrimination paradigm in which stimuli are presented at a fixed periodic rate eliciting frequency-locked responses in the EEG signal (Rossion, 2014). Available research has shown this technique to be efficient in providing an objective neural index of subtle visual discrimination in the domain of face perception in adults (Rossion, 2014; Rossion et al., 2020) and infants (Baccolo et al., 2023; De Heering & Rossion, 2015; Peykarjou, 2022). It consists of presenting a range of stimuli at a fixed periodic frequency, which generates a periodic change in voltage amplitude in the EEG signal at the same stimulation frequency. Following the logic of the D-MOP, human faces could be presented at a fixed base rate of six images per second (6 Hz) periodically interrupted by doll-like faces every fifth stimulus. This would generate an oddball frequency of 1.2 Hz (6 Hz/5), corresponding to the periodic appearance of doll-like faces. A significant EEG response at this oddball frequency would index infants’ or adults’ capacity to differentiate doll-like from human faces. Critically, blocks could manipulate group membership by presenting exclusively White or Black faces, allowing researchers to test whether discrimination of the doll-like oddball is enhanced for ingroup compared to outgroup faces, as demonstrated in the original D-MOP paradigm. Interestingly, this design would further allow developmental comparisons among 3- and 4-month-old infants, who are not yet expected to show robust own- versus other-ethnicity differentiation, with 9- to-12-month-old infants and adults, for whom such distinctions are more likely to emerge.

Another methodology that could prove particularly interesting to adopt in the current context and that is shared across ages, allowing comparisons between children and adults, is facial mimicry. Facial mimicry, defined as the tendency to automatically synchronize facial expressions with those of another person, is already present at birth (Meltzoff, 1999) and is modulated by the observation of emotional faces as early as 5 months of age (Isomura & Nakano, 2016; Kaiser et al., 2017), continuing through the preschool years (Geangu et al., 2016). Mimicry appears to have evolved to foster affiliation, although this tendency is constrained by social similarities between the mimicker and the mimickee (Hess & Fischer, 2017). Existing studies have demonstrated that facial mimicry, as assessed through surface electromyography (sEMG), is more likely when adults (Likowski et al., 2008) or infants (Skinner et al., 2020) have positive rather than negative attitudes toward targets or when the targets speak the infants’ native rather than a different language (De Klerk et al., 2019). In addition, infants tend to overtly imitate social but not inanimate agents, such as mechanical devices or mindless robots (Meltzoff, 1995). In a similar vein, adults displayed weaker mimicry reactions toward an android face expressing an emotion the more it was perceived as mindless compared to a real human face (Hofree et al., 2014). Among adults, often dehumanized targets such as sexually objectified women were also mimicked less compared to their fully dressed non-objectified counterparts (Ruzzante & Vaes, 2024b). As such, facial emotional mimicry assesses the extent to which we engage with others. Failing to mentalize others because we do not want to know them (e.g., outgroup members) or because we do not think they have a mind (e.g., robots), is expected to interfere with this spontaneous mechanism. In other words, less spontaneous mimicry might be directly related to processes of dehumanization (Harris & Delgado, 2025).

These premises make facial emotional mimicry and sEMG a particularly interesting methodology to study the emergence of dehumanization biases across the life span. Comparing mimicry reactions toward ingroup and outgroup faces that differ in ethnicity among infants, pre-school children, and adults might provide unique insights into the inception of dehumanized perceptions. Moreover, one could also add doll-like ingroup and outgroup faces to directly manipulate the mindfulness of the targets and verify whether outgroup faces are mimicked to a similar extent as the mindless doll-like faces. Such a finding would overlap with the fading of the human–object divide that is central in our process account of dehumanization. With preschool children and adults, individual differences in mind-attribution skills could also be assessed and correlated with the spontaneous mimicry behavior of participants.

## 8. Conclusions

Research on dehumanization has flourished over the last three decades, culminating in our knowledge of the meaning of the phenomenon, the many ways it can manifest itself, and the fundamental reasons that drive its occurrence. The current review provides a brief overview of the main developments within this area of research while recognizing that previous reviews have catalogued the various forms of dehumanization (e.g., subtle vs. blatant; animalistic vs. mechanistic) but stopped short of analyzing the processes that drive dehumanized perceptions (but see Harris & Delgado, 2025, for a recent exception). Therefore, a process account of dehumanization was introduced, and some initial research findings were presented that seem to confirm the main tenets of this new framework. Rooted within our capacity to reason about others’ minds, this process account of dehumanization enhances our understanding of the cognitive and neurological processes that underlie dehumanized perceptions. The introduction of a novel methodology that directly compares people’s neural and behavioral reactions to mindless, doll-like and mindful, human faces, goes beyond semantic measures that try to capture humanness in the attribution of a limited set of human characteristics. As a result, we were able to formulate some constructive responses to some of the challenges and criticisms that have targeted characteristic-based measures of dehumanization.

The process account of dehumanization allowed us to establish meaningful connections with related fields and phenomena, such as face and emotion recognition, empathy and person perception, all of which contribute to a full understanding of the other. The connections with other fields help to identify both the relevant processes and methodologies and open the possibility of proposing a research agenda testing the developmental mechanisms that drive the emergence of dehumanized perceptions. We believe this research agenda will be crucial for unveiling some of the necessary cognitive abilities and processes that underly dehumanization, providing us with fundamental information about its emergence and inner workings.

## Data Availability

No new data were created or analyzed in this study.
